# Annexin A1 and A2: Roles in Retrograde Trafficking of Shiga Toxin

**DOI:** 10.1371/journal.pone.0040429

**Published:** 2012-07-06

**Authors:** Lionel Tcatchoff, Sofia Andersson, Audrun Utskarpen, Tove Irene Klokk, Sigrid S. Skånland, Sascha Pust, Volker Gerke, Kirsten Sandvig

**Affiliations:** 1 Department of Biochemistry, Centre for Cancer Biomedicine, Institute for Cancer Research, The Norwegian Radium Hospital, Oslo University Hospital, Montebello, Oslo, Norway; 2 Department of Molecular Biosciences, University of Oslo, Oslo, Norway; 3 Institute for Medical Chemistry, Center for Molecular Biology of Inflammation, University of Münster, Münster, Germany; University of Nebraska Medical Center, United States of America

## Abstract

Annexins constitute a family of calcium and membrane binding proteins. As annexin A1 and A2 have previously been linked to various membrane trafficking events, we initiated this study to investigate the role of these annexins in the uptake and intracellular transport of the bacterial Shiga toxin (Stx) and the plant toxin ricin. Once endocytosed, both toxins are retrogradely transported from endosomes to the Golgi apparatus and the endoplasmic reticulum before being targeted to the cytosol where they inhibit protein synthesis. This study was performed to obtain new information both about toxin transport and the function of annexin A1 and annexin A2. Our data show that depletion of annexin A1 or A2 alters the retrograde transport of Stx but not ricin, without affecting toxin binding or internalization. Knockdown of annexin A1 increases Golgi transport of Stx, whereas knockdown of annexin A2 slightly decreases the same transport step. Interestingly, annexin A1 was found in proximity to cytoplasmic phospholipase A2 (cPLA_2_), and the basal as well as the increased Golgi transport of Stx upon annexin A1 knockdown is dependent on cPLA_2_ activity. In conclusion, annexin A1 and A2 have different roles in Stx transport to the *trans*-Golgi network. The most prominent role is played by annexin A1 which normally works as a negative regulator of retrograde transport from the endosomes to the Golgi network, most likely by complex formation and inhibition of cPLA_2_.

## Introduction

Numerous toxins take advantage of the mammalian cellular machinery to enter and reach their target within the cell. The bacterial Shiga toxin (Stx) and the plant toxin ricin are examples of such toxins, and do therefore represent valuable tools to study endocytic and intracellular transport mechanisms (for recent reviews, see [1], [2]). These toxins are built up of an A and a B moiety, where the latter facilitates the binding to cellular receptors, the glycosphingolipid globotriaosylceramide (Gb3) for Stx and terminal galactose residues of glycolipids and glycoproteins for ricin. After entering the cell by endocytosis, the toxins are sorted in the early endosomes and are either recycled, destined for lysosomal degradation or transported retrogradely to the Golgi apparatus and the endoplasmic reticulum (ER). The enzymatically active part of the A subunit is then translocated from the ER to the cytosol where it exerts its cytotoxic effect by inactivating ribosomal function leading to an inhibition of protein synthesis [2], [3].

During the past years, a variety of studies have resulted in the identification of proteins that are necessary for the highly regulated transport events used by toxins. These include clathrin and its binding partners [4]–[7], sorting nexins [5], [8]–[11] as well as different lipids [12], [13]. Moreover, binding of Stx has been shown to trigger activation of various signalling molecules, including protein kinase Cδ, Syk and p38 [14]–[16].

Annexins, a family of structurally related Ca^2+^-binding proteins [17], are of interest in the search for candidate proteins that take part in the uptake and transport of Stx and ricin. Annexins comprise a conserved C-terminal core domain interacting with membrane phospholipids and a variable N-terminal domain, which is responsible for the specific functions of individual annexins. The N-terminal part of annexin A1 contains three possible phosphorylation sites while annexin A2 contains phosphorylation sites which are presumable targets for PKCα and Src kinases. Both N-terminal domains also harbour interaction motifs for proteins of the S100 family, S100A11 and S100A10 (also named p11) for annexin A1 and A2 respectively [18]. Annexin A1 and A2 have both been shown to function in different steps along the endocytic pathway [19], [20]. Annexin A1, a highly abundant cellular protein, has been reported to take part in the epidermal growth factor-induced formation of internal vesicles within multivesicular bodies (MVBs). After EGF stimulation, the number of MVBs and internal vesicles in MVBs is increased. Futter and coworkers showed that during this process, annexin A1 serves as a substrate for the EGFR tyrosine kinase [21]. The phosphorylation of tyrosine in position 21 appears critical for the EGF-induced inward vesiculation process during MVB maturation, while the biogenesis of MVBs itself does not require annexin A1 [22]. Annexin A2 is present on early endosomes [23]–[25] and has been found attached to clathrin coated vesicles [26]. It has also been reported that expression of mutated annexin A2 or annexin A2 siRNA treatment induces a redistribution of transferrin-positive endosomes from the periphery to a more central region of the cytoplasm or to the perinuclear area [24], [27]. Furthermore, Mayran and coworkers showed that knocking down annexin A2 by siRNA inhibits transport of EGF from early to late endosomes. Their data indicate that annexin A2 is required for the formation of MVBs [28]. Thus, interaction of annexin A1 and A2 with membrane regions has been extensively studied; however, their possible role in endosome to Golgi transport has so far not been investigated.

Several studies have shown that the α isoform of cytoplasmic phospholipase A2 (cPLA_2_α) regulates the structure and function of the Golgi apparatus (for review see [29]){Leslie, 2010 128/id}. Furthermore, using inhibitors of cPLA_2_, a general role of cPLA_2_ in the transport of cargos from early or recycling endosomes via tubule formation has also been described [30], [31]. cPLA_2_ releases fatty acids and thereby influences membrane curvature and tubulation. Importantly, several reports show an interaction in vitro of cPLA_2_α with annexin A1 [32] as well as with S100A10 [33], the complex S100A10-annexin A2 [34] and a fusion protein containing S100A10 and annexin A2 [32]. In addition, complex formation between annexin A1 and cPLA_2_α has an inhibitory effect on cPLA_2_α activity. More recently, it has been shown that in confluent endothelial cells, inactive cPLA_2_α in complex with annexin A1 accumulates at the Golgi apparatus. This Golgi association was absent in non-confluent endothelial cells and could not be observed in HeLa cells [35].

In the present study we analyzed whether annexins A1 and A2 participate in the retrograde transport of toxins. We show that both annexin A1 and A2 have a role in the retrograde transport of Stx but not of ricin. Interestingly, these two members of the annexin family exert different effects on the retrograde transport of Stx to the *trans*-Golgi network. Knockdown experiments indicate that annexin A1, possibly by complexing and inhibiting cPLA_2_, works as a negative regulator of retrograde Stx transport from endosomes to the Golgi, whereas annexin A2 slightly promotes this route of Stx trafficking.

## Results

### Role of annexin A1 and A2 in the regulation of Stx transport to the Golgi apparatus

**Figure 1:**
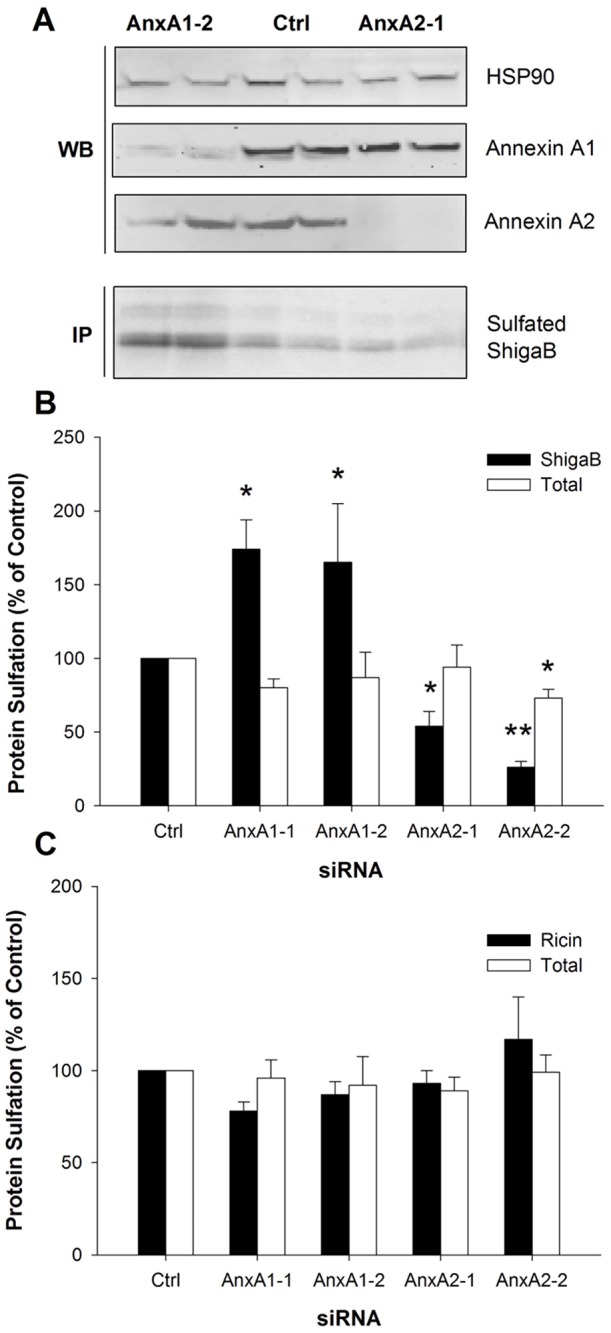
ShigaB transport to the Golgi is regulated by annexin A1 and A2. HeLa cells were transfected with control, annexin A1 or A2 siRNA for 72 h. For sulfation measurements, cells were starved in the presence of radioactive sulfate for 3 h. ShigaB-sulf2 or Ricin-sulf1 was then added and the incubation proceeded for an additional one or two hours, respectively. Cells were lyzed, ShigaB or ricin immunoprecipitated, separated by electrophoresis and analyzed by autoradiography. The protein knockdown level was investigated in total cell lysates by immunoblotting. (A) Cell lysates were analyzed by western blotting (upper panel) with the indicated antibodies demonstrating protein knockdown of annexin A1 and A2 by the indicated siRNA oligos. Hsp90 represents loading control. Autoradiography (lower panel) showing results from the corresponding sulfation experiment. (B) and (C) Quantative data from protein sulfation for ShigaB and ricin respectively, plotted as percentages of control values. Quantifications of sulfation are the average of 3–8 independent experiments, each performed in parallel, error bars indicating standard error of the mean; *p<0.05, **p<0.005 indicate statistically significant change.

Given the reported involvement of annexin A1 and A2 in endocytic transport we first monitored the retrograde movement of toxin to the Golgi apparatus after knockdown of annexin A1 and A2 using small interfering RNA. For this purpose, we took advantage of the post-translational sulfation reaction catalyzed by the tyrosyl sulfotransferases exclusively present in the trans-Golgi network [36], [37]. Modified Stx and ricin molecules were used. ShigaB-sulf2 [38] consists only of the Stx B-chain with two available tyrosine sulfation sites added, whereas ricin-sulf1 [39] is the intact toxin with one sulfation site in the A-chain. Real-time RT-PCR indicated that mRNA levels of annexin A1 and A2 were strongly reduced by siRNA targeting the corresponding mRNA (figure S1). As expected, annexin A1 and A2 protein expression was also severely reduced by siRNA treatment, as assessed by Western blotting (Figure 1A, top panel). Moreover, levels of annexin A1 mRNA and protein were not reduced by siRNA treatment against annexin A2 and vice versa, showing the specificity of the siRNA used. Also, knockdown of one of the genes did not induce any compensatory up-regulation of the other. Annexin A1 or A2 protein knockdown both influenced the level of ShigaB sulfation, but surprisingly in opposite directions compared with control cells. Indeed, as shown in figure 1A (lower panel) and B, Stx sulfation in HeLa cells was increased to 174±20% and 165±40% for the AnxA1-1 and AnxA1-2 siRNA oligo, respectively. For HEp-2 cells the corresponding values were 153±28% and 178±20% (figure S2). In contrast, knockdown of annexin A2 resulted in an Stx sulfation level of 54±10% for the AnxA2-1 and 26±10% for the AnxA2-2 siRNA oligo, respectively, in HeLa cells (Figure 1B). Again, the results were similar for HEp-2 cells, with a reduction to 68±5% and 78±6% (figure S2). Interestingly, no significant change was observed for ricin sulfation in HeLa cells after knockdown of annexin A1 or A2 (Figure 1C), indicating that the roles of annexin A1 and A2 are specific for Stx trafficking. In order to confirm that the detected changes in sulfation of the toxin constructs were not due to a general alteration of sulfation in the cell, the sulfation levels of total cellular proteins were investigated in parallel. A modest decrease of total sulfation (73±6%, – *p* = 0.015) was only observed for cells treated with AnxA2-2 siRNA in ShigaB sulfation experiments (Figure 1B) while there was no effect for the other treatments or in the ricin experiment (Figure 1C).

**Figure 2:**
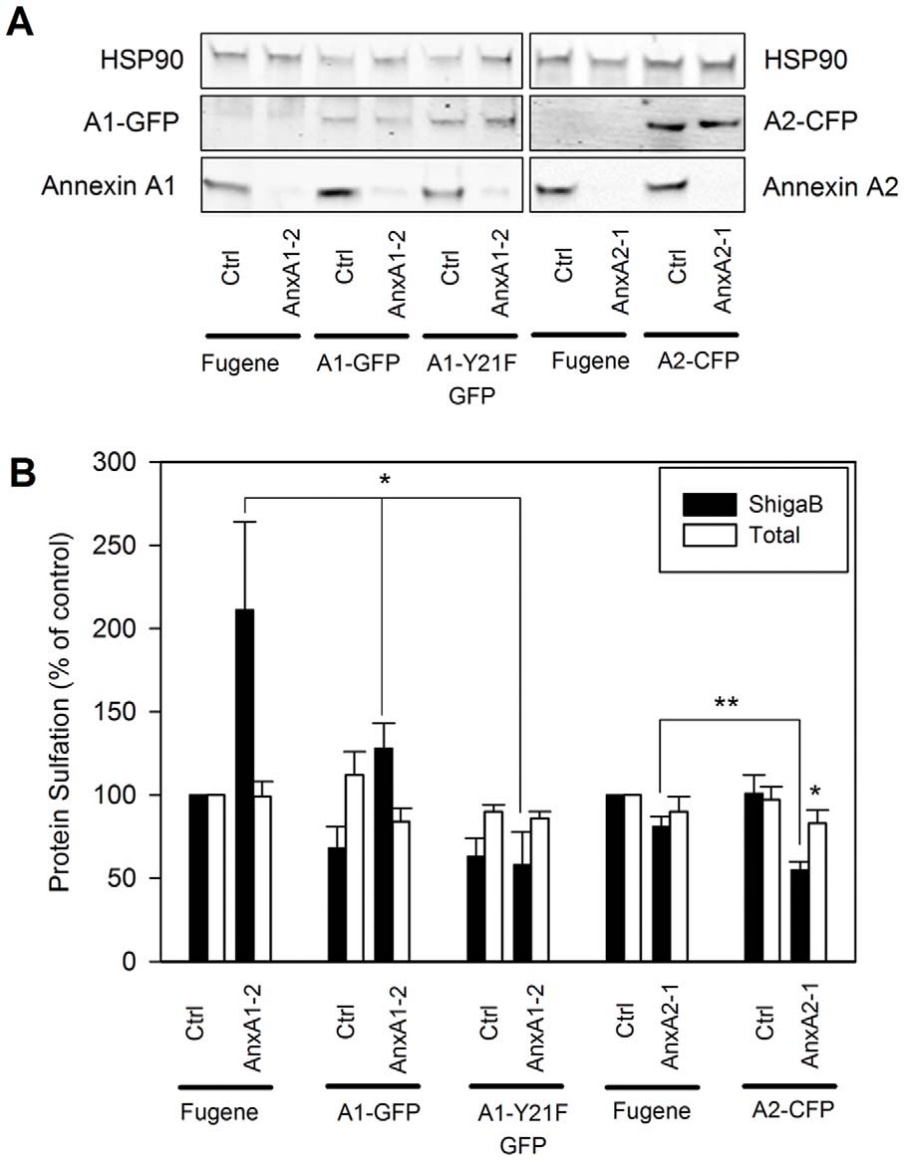
Rescue of phenotype by over-expression of wild type annexins. HeLa cells where transfected with indicated siRNA and subjected for 24 h either to Fugene treatment as control, A1-GFP, A1-Y21F-GFP or A2-CFP transfection as indicated. (A) Western blots of cells lysates for the conditions stated under each lane, stained with the indicated antibody and showing expression of constructs and/or endogenous annexins for one representative experiment (duplicates are not shown here for more clarity). (B) Quantative data from ShigaB sulfation (black bars) and total protein sulfation (white bars) are plotted as an average percentage of control values for at least 3 independent experiments, each performed in parallel, error bars indicating standard error of the mean; *p<0.05 indicates statistically significant change.

To verify that the cellular phenotypes resulting from annexin A1 and A2 depletion were specific, we performed rescue experiment using plasmids expressing wild type annexin A1 or A2 fused to GFP and CFP respectively. Plasmids were introduced to the cells 48 h after siRNA treatments and expression allowed to occur for 24 h before sulfation experiments were performed. Even if the plasmids used are not siRNA resistant, Western blots shown in figure 2A reveal a comparable expression level of each fusion protein even in siRNA treated cells, while expression of the corresponding endogenous protein is strongly reduced by the treatment. Protein sulfation quantification plotted in figure 2B indicates that overexpression of annexin A1-GFP in cells where annexin A1 had been depleted, strongly reduced the elevated ShigaB sulfation from 211±53% of control to 128±15%, demonstrating an almost complete rescue. On the other hand, annexin A2-CFP overexpression in annexin A2 depleted cells leads to a further decrease in ShigaB sulfation to 55±5% instead of the expected rescue. The decrease in ShigaB sulfation correlates with a small but significant decrease in total sulfation, down to 83±8% (*p* = 0.01), compared to control cells. Total sulfation was not affected when annexin A2-CFP was expressed in cells which had not been treated with annexin A2 targeting siRNA, indicating that the double treatment seems to be toxic for the cells. This was not investigated in further detail since we chose to focus on the ability of annexin A1 knockdown to increase Golgi transport.

A possible explanation for the increased sulfation of ShigaB after annexin A1 depletion could be that an alternative route, to for instance lysosomes, is inhibited when annexin A1 is eliminated, thereby redirecting more Stx towards the Golgi apparatus. It has indeed been reported that depletion of annexin A1 inhibits EGF-induced formation of internal vesicles during MVB formation [21], [22] and that a normal MVB phenotype is rescued by an annexin A1-GFP construct but not by a Y21F mutant of annexin A1 which cannot be phosphorylated by the EGF receptor [22]. Although our cells had not been stimulated by addition of EGF, they were grown in serum-containing medium and we therefore tested the effect of overexpressing annexin A1-Y21F-GFP. As shown in figure 2, overexpression of A1-Y21F-GFP induces a significant reduction of ShigaB sulfation both in the absence and presence of annexin A1 knockdown, indicating that phosphorylation of tyrosine 21 is not needed for the role of annexin A1 in controlling ShigaB sulfation levels.

Due to the results of these experiments we became interested in finding out whether Stx might colocalize with any of the investigated annexins. Annexin A1 staining turned out to be very intense in the nuclei, strong at the plasma membrane and with some cytosolic labeling (figure S3). Annexin A2 showed a similar distribution, although it was absent from the nucleus. Incubation with StxB-K3 prelabeled with Alexa 488, prepared as described previously [12], resulted in a colocalization between Stx and both annexin A1 and A2 at regions of the plasma membrane, and partial intracellular colocalization was also observed (figure S3).

**Figure 3:**
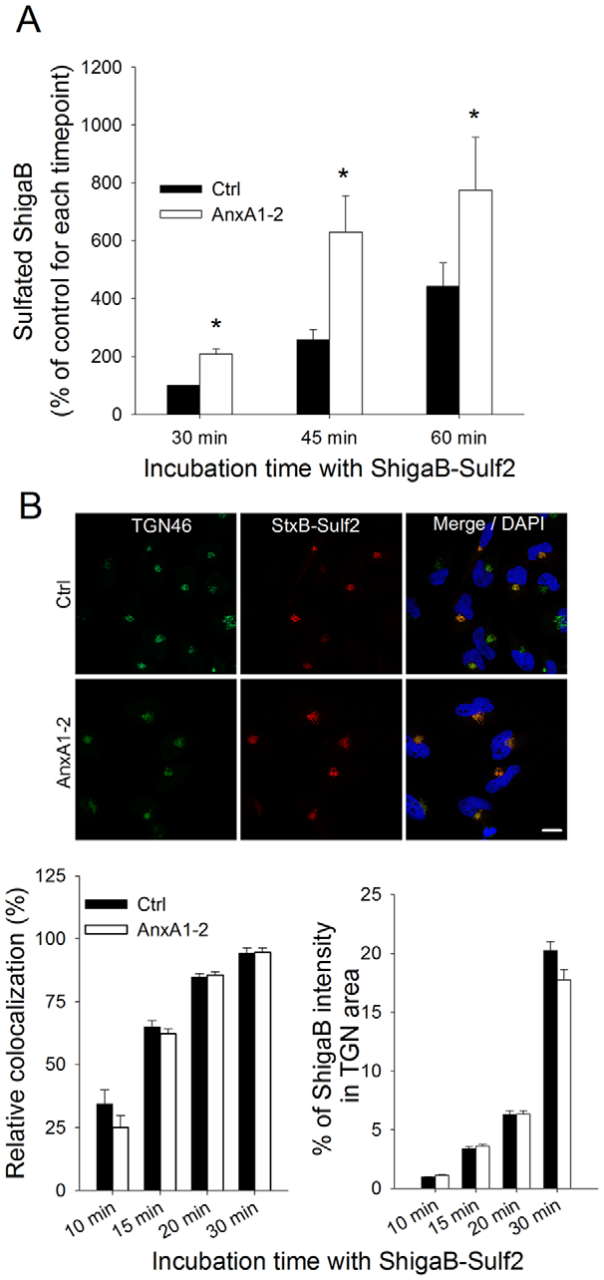
Time course of ShigaB sulfation in annexin A1 or A2 depleted cells. (A) HeLa cells transfected with control or annexin A1 siRNA for 72 h were analyzed for the amount of ShigaB being sulfated at different time points after addition of StxB. Sulfation data are plotted as percentages of the amount of sulphated ShigaB after 30 min incubation with control siRNA treated cells. Black and white bars represent sulfation of ShigaB in control and annexin A1 siRNA treated cells, respectively. Data presented are the average of 3–4 independent experiments, each performed in parallel, error bars indicating standard error of the mean, *p<0.05; **p<0.005 indicates statistically significant change. (B) After the same treatment as in (A), cells were fixed and stained with antibodies against TGN46 and ShigaB. Top panel shows representative confocal pictures for 30 min incubation with StxB, scale bars 20 μm. Left graphic shows quantification of amount of ShigaB colocalized with TGN46. In the right graphic, mean intensity of ShigaB in the Golgi area for the same representative experiment is plotted as percentage of mean intensity in the whole cell. Data presented are the average of at least 35 cells per condition. Quantifications where obtained with ImageJ software, error bars indicating standard error of the mean.

To examine in further detail the role of annexin A1 in Shiga toxin trafficking, we performed a time course of the sulfation of ShigaB. Notably, as shown in figure 3A, Shiga sulfation was increased from 2–2.5 times at each time point, suggesting that annexin A1 knockdown resulted in more ShigaB entering the Golgi region. As expected, confocal analysis revealed that colocalization of ShigaB and TGN46 increased with time, as shown in figure 3B. Quantification of intensity also indicates a time-dependent increase in the signal caused by ShigaB in the *trans*-Golgi network (as % of total ShigaB) for control and cells depleted of annexin A1 (Figure 3B), while TGN signal is not affected (data not shown). It is worth to note that the percentage of ShigaB in the Golgi apparatus appears less than what it seems from the colocalization values, due to the threshold applied during the colocalization analysis leading to exclusion of weak ShigaB signals. In both cases, knockdown of annexin A1 does not appear to affect the kinetics of ShigaB trafficking since neither the colocalization with TGN46, nor the proportion of ShigaB in the Golgi at the different time points, are affected by annexin A1 knockdown. It is therefore likely that the toxin is not accumulating in the TGN compartment after being sulfated, but continues retrogradely to the endoplasmic reticulum.

### Golgi markers and M6PRs are not affected by Annexin A1 depletion

Our sulfation data clearly indicate that Stx transport to the Golgi is affected when annexin A1 is depleted. Endogenous proteins, such as mannose-6 phosphate receptors (MPRs) have also been shown to travel retrogradely from endosomes to the Golgi network. Immunofluorescence and electron microscopy imaging have shown that both the cation dependent (46 kDa) and the cation independent (300 kDa) MPR are localized to perinuclear late endosomes in NRK and BS-C-1 cells at steady state, with low levels present in other compartments [40], [41]. Therefore, we analyzed the distribution of MPRs in relation to the early endosomal marker EEA1, the *cis*-Golgi marker giantin as well as TGN46 in annexin A1 depleted cells. None of these markers are relocalized by knockdown of annexin A1, and as shown in figure S4, no redistribution was observed for MPR46 or MPR300 relatively to TGN46 positive structures in the depleted cells. Quantative colocalization analysis also showed that the major fraction of both MPR46 and MPR300 were localized to the TGN in HeLa cells regardless of annexin knock-down. We also did not observe any change in colocalization of either MPRs with EEA1 and giantin in depleted versus control cells (data not shown). Since annexin A1 knockdown changes the ShigaB sulfation, we decided to study the localization of the sulfotransferases TPST1 and TPST2. Earlier studies have shown that the redistribution of these sulfotransferases caused by Exo2 treatment, could be linked to an altered Stx sulfation [42]. In agreement with previous studies [6], [42], we found that over 80% of transiently expressed TPST2 colocalized with TGN46. Importantly, as shown in figure S4B, the relative distribution of TPST2 and TGN46 remained unchanged after knockdown of annexin A1. This is also true for another Golgi marker, giantin. Moreover, there is still no colocalization of TPST2 with the early endosome marker EEA1 when annexin A1 is knocked down (data not shown). Similar results were obtained for the sulfotransferase isoform TPST1, supporting the idea that annexin A1 participate in Stx transport to the Golgi without affecting the Golgi apparatus itself and its enzyme repertoire. Also the fact that the total sulfation and the ricin sulfation (Figure 1B and 1C) are not affected by any knockdown strongly supports the idea of a specific role of annexins in Stx trafficking.

### Stx binding and endocytosis are independent of Annexin A1

To investigate if the changes in the Stx sulfation levels were due to alterations in the binding or endocytosis of the toxin, we followed the uptake of Stx in HeLa cells exhibiting reduced annexin A1 protein levels using an electrochemical method, allowing us to distinguish between internalized and total cell associated Stx [15]. Measurements done at 10, 20 and 30 min after addition of toxin revealed no change in the amount of endocytosed or total cell associated Stx after knockdown of annexin A1 compared with control cells (figure S5).

### The effect of annexin A1 knockdown on Stx transport is impaired by inhibition of PKCδ or PLA2

**Figure 4:**
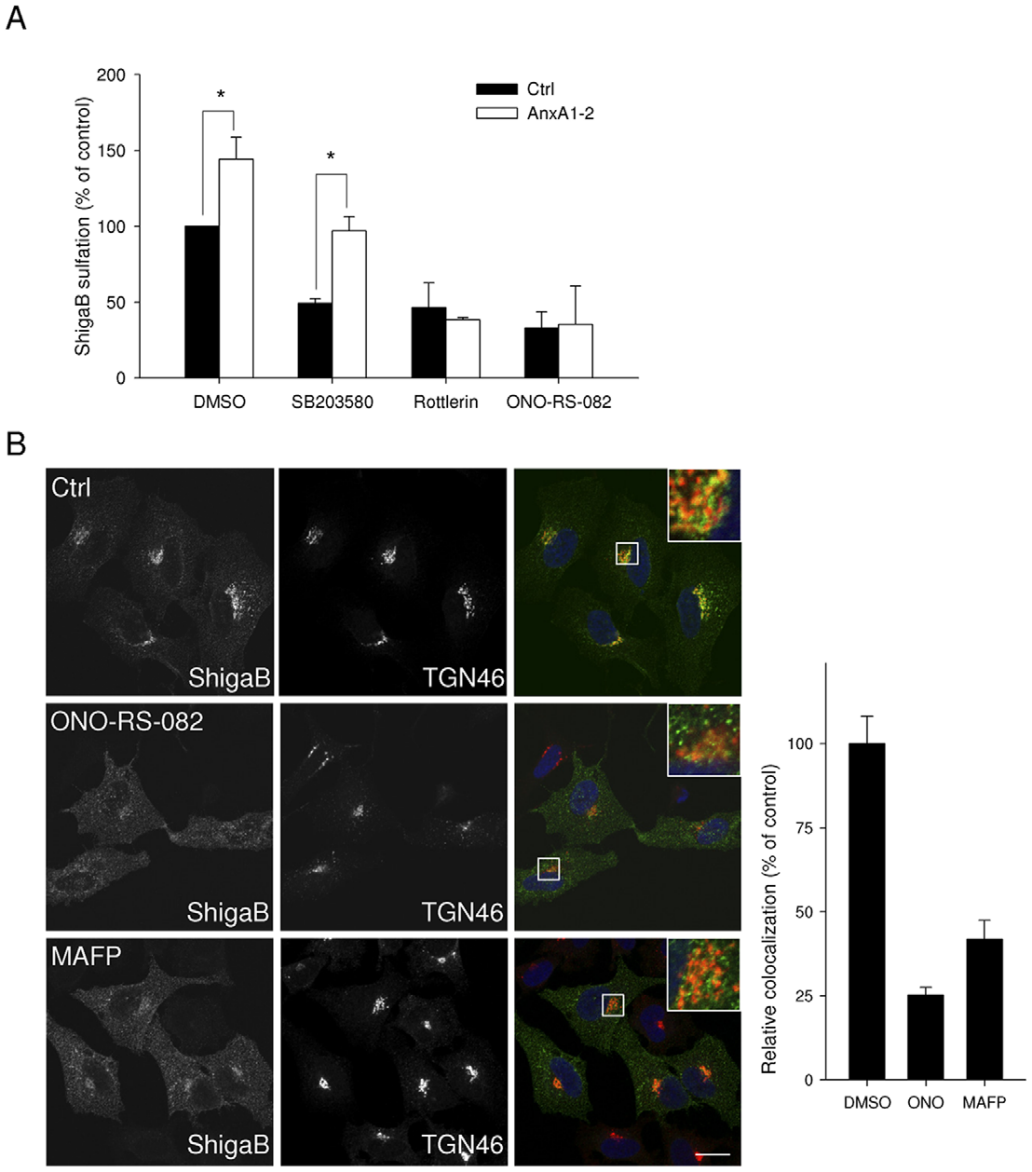
Stx transport in annexin A1 depleted cells is regulated by PKCδ and PLA2. (A) Golgi transport of Shiga toxin was evaluated as described in materials and methods by quantification of sulfated ShigaB in HeLa cells transfected with siRNA against annexin A1 or non targeting siRNA, pretreated with the indicated inhibitors. Data from Stx sulfation are plotted as percentages of the value obtained for HeLa cells transfected with control siRNA and treated with DMSO. The white and black bars represent ShigaB-sulf2 sulfation for control and annexin A1 knockdown cells respectively. Data presented are the average of 3 independent experiments, each performed in parallel, error bars indicating standard error of the mean. *p<0.05 indicates statistically significant change between annexin A1 knockdown cells and the corresponding control siRNA treated cells. (B) After treatment with either 5 µM ONO-RS-082 for 30 min or 30 µM MAFP for 1 h, HeLa cells were incubated for 30 min with ShigaB before fixation and staining as indicated in the materials and methods section with antibodies against TGN46 and ShigaB. Panel shows representative confocal pictures, scale bars 20 μm. Graphic shows quantification of amount of ShigaB colocalized with TGN46 in one representative experiment plotted as percentage of control condition. Data presented for one representative experiment (n = 3) are the average of at least 30 cells per condition. Quantifications were obtained with Zen 2009 software from Zeiss, error bars indicating standard error of the mean.

**Figure 5:**
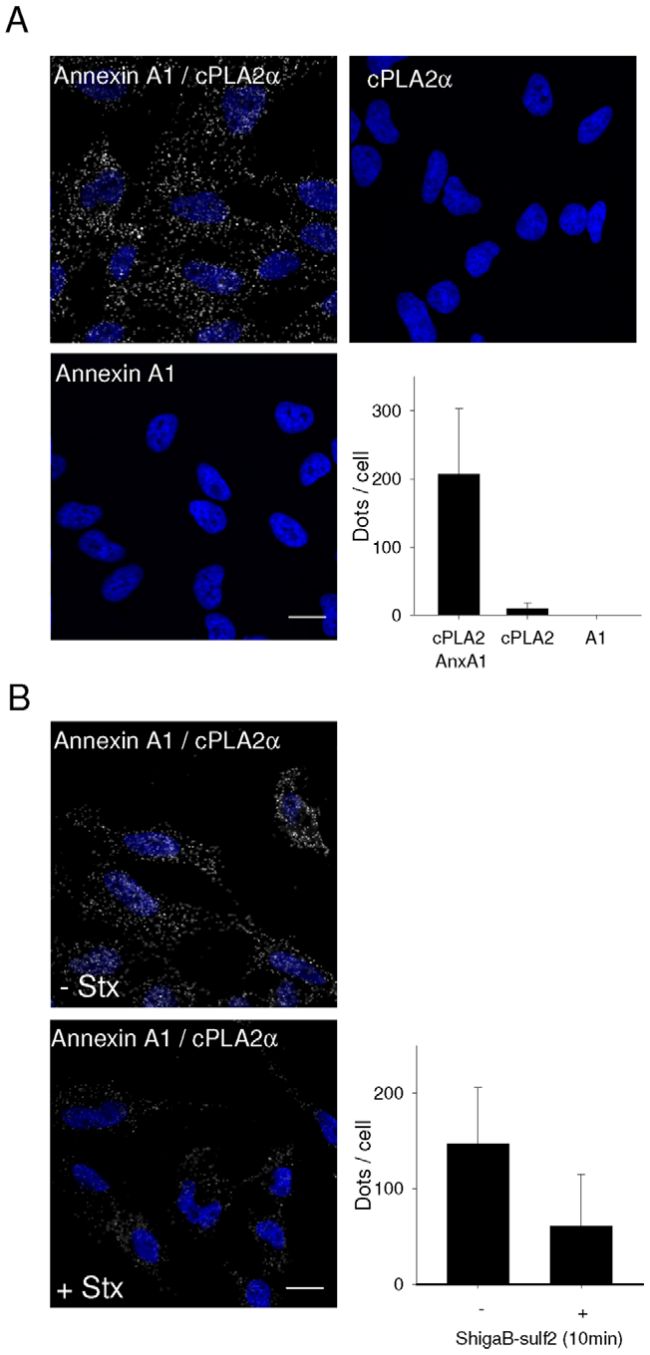
Close proximity of Annexin A1 and cPLA2α in HeLa cells. (A) Close proximity of annexin A1 with cPLA_2_α was evaluated using proximity ligation assay from Duolink. Fixed and permeabilized cells were incubated with the indicated primary antibodies by pair or alone as negative control. Scale bar is 20 μm. Dots per cell were automatically counted using ImageJ software and the data presented in the diagram is the average of at least 40 cells per condition in one representative experiment (n = 3), error bars indicating standard deviation. Values for negative controls were 10.3±2.7 and 0.3±0.1 dots per cell for cPLA_2_ α and annexin A1 antibodies respectively. (B) Before proximity ligation assay, cells were washed and incubated for 30 min in Hepes buffered medium. They were subsequently incubated for 10 min with ShigaB before staining with annexin A1 and cPLA_2_α antibodies in combination. Scale bar is 20 μm. Interaction events where evaluated as in A. Data presented are the average of at least 40 cells quantified per condition for one representative experiment (n = 3), error bars indicating standard deviation.

With regards to the strong effect of annexin A1 knockdown on Stx transport, we then focused on its possible mechanisms of action. It has been shown that Stx regulates its own transport through activation of p38 and PKCδ [15], [16]. To investigate if the role of annexin A1 in Stx transport from early endosomes to the Golgi apparatus is related to these kinases and signalling events, we compared sulfation of Stx in cells treated with non-targeting or annexin A1 targeting siRNA in the absence or presence of inhibitors of the MAPK p38 and PKCδ. As shown in figure 4A, the strong increase in sulfation of ShigaB in annexin A1-depleted cells is still observed in the presence of SB203580, an inhibitor of p38 (from 49±3% to 97±10% of control, – *p* = 0.018), suggesting that the increased Golgi transport occurs independently of p38 activity. Interestingly, we no longer observed increased ShigaB sulfation following annexin A1 knockdown when cells were incubated in the presence of rottlerin, an inhibitor of PKCδ. It should be noted that we observed no significant change in the total protein sulfation in the same experiments (data not shown). This result indicates that the increased transport of Stx initiated by the lack of annexin A1 involves the activity of PKCδ. In other words, PKCδ and annexin A1 act in the same pathway with regards to Stx retrograde transport. Since annexin A1 has been reported to inhibit cPLA_2_α activity which may facilitate membrane tubulation and transport to the Golgi, we evaluated the role of cPLA_2_ in retrograde transport of Stx. Treatment with ONO-RS-082, an inhibitor of cPLA_2_, induced a strong reduction in Stx sulfation in both annexin A1 targeting siRNA treated cells and control cells (figure 4A), suggesting that cPLA_2_ and annexin A1 act on the same pathway. The involvement of cPLA_2_ in the retrograde transport of Shiga toxin was confirmed by the use on another cPLA_2_ inhibitor, MAFP, which resulted in a dose-dependent reduction in ShigaB sulfation (figure S6). Interestingly, the colocalization between TGN46 and Shiga toxin after 30 min is reduced to 25 and 41% compared to control after ONO-RS-082 or MAFP treatment, respectively, as shown in figure 4B, indicating that upon inhibition of cPLA_2_, Shiga toxin trafficking to the Golgi is impaired. Altogether, these data are in line with an interaction between cPLA_2_α and annexin A1 that has been demonstrated in vitro [32] and in pull down experiments with lysates from normal human keratinocytes [43]. However, although it was reported that cPLA_2_α and annexin A1 were localized to the Golgi apparatus in human umbilical vein endothelial cells, this could not be observed in HeLa cells [35]. While cPLA_2_α is able to relocalize depending on cell context, for example confluence status, presence of growth factors or the induction of secretory pathways [35], [44], [45], its canonical distribution appears to be mainly cytoplasmic [35], [46]–[48]. To study a possible interaction between cPLA_2_α and annexin A1 in HeLa cells, we used an *in situ* proximity ligation assay (Duolink) [49]. This system has been used in several studies to demonstrate proximity of protein partners. For instance, it has been used to demonstrate the proximity of the sortilin-related receptor and lipoprotein lipase during trafficking of lipoprotein lipase from the TGN to endosomes [50] and to demonstrate proximity between cPLA_2_α and EHD1 [51]. The assay gives a positive signal or dot on confocal pictures when the distance between two molecules is less than 40 nm. To evaluate the specificity of the assay, we confirmed that overexpressed GFP and annexin A1, both cytoplasmic proteins, did not give any proximity signals (figure S7). Moreover, in negative controls, using only one antibody as a probe, very few spots were detected, 10 and less than 1 spot per cell in average for cPLA_2_α and annexin A1, respectively (figure 5A). As shown in figure 5A, when using antibodies against annexin A1 and cPLA_2_α together, quantification revealed ∼200 dots per cells, indicating close proximity between the two proteins. Interestingly, the number of interaction events appears reduced from ∼150 to 60 dots per cell if the cells are incubated for 10 min with ShigaB prior to staining (figure 5B), showing that the annexin A1/cPLA_2_α complex is labile and affected by the transported cargo.

## Discussion

In the present study we provide evidence for a role of annexin A1 and A2 in the retrograde transport of Stx to the Golgi apparatus. Importantly, we discovered different roles for the two annexins: knockdown of annexin A1 increased the transport of Stx to the Golgi apparatus, whereas knockdown of annexin A2 seemed to decrease this transport. As observed earlier [9], [15], [16], [52], transport of the plant toxin ricin to the Golgi does not require the same machinery as that involved in Stx transport, illustrated by the fact that knockdown of annexin A1 or A2 did not affect ricin transport. This may be due to the ability of ricin to bind various glycoproteins and glycolipids, a property that may allow ricin to use various pathways to a given destination, in this case the Golgi apparatus.

The increasing number of studies showing that depletion or overexpression of specific proteins interferes with the transport of Stx, points out the complexity of toxin transport [2], [51], [54]. Since Shiga toxin can be internalized through both clathrin-dependent and -independent mechanisms [4], [55], and annexin A1 has been reported to interact with μ subunits of the clathrin assembly protein complex AP-2 [56], annexin A1 could be required for the internalization process of Stx. However, utilizing different experimental approaches we showed here that plasma membrane binding and endocytosis of Stx were not affected by annexin A1 protein depletion.

Surprisingly, we observed that depletion of annexin A1 increases the level of ShigaB sulfation, suggesting that annexin A1 normally works as a negative regulator of the toxin transport. It has been reported that depletion of annexin A1 inhibits EGF-induced formation of internal vesicles during MVBs formation [21], [22]. The commonly accepted model for this is that the activated EGF receptor phosphorylates annexin A1 at tyrosine 21, which in turn becomes more sensitive to proteolysis. Degradation of annexin A1 linked to the membrane at the rim of budding internal vesicles leads to fusion of opposite membranes and thereby facilitates vesicle formation [18]. The possibility that an alternative route, to for instance lysosomes, is inhibited when annexin A1 is eliminated, thereby redirecting more Stx towards the Golgi apparatus, has been investigated here and could be excluded. Indeed, overexpression of an annexin A1 mutant of Tyr21 shown to be unable to restore EGF-induced formation of internal vesicles, is rescuing a normal Stx transport to the Golgi. Altogether our data suggest a more direct role of annexin A1 in endosomes to Golgi transport that has not been described to date.

In addition to toxins, many endogenous proteins such as the MPRs also travel retrogradely from early endosomes to the Golgi network. By studying these molecules, evidence for different transport routes between endosomes and the Golgi have emerged [2], [53]. There are several similarities in the trafficking of MPRs and Stx. For instance, depletion of components of the retromer complex interferes with the retrograde transport of both cargos [5], [6], [8], [11], [57]–[59]. However, some differences also exist in the regulation of Stx and MPR transport from early endosomal compartments to the TGN. Among others, Rab11 has been reported to be involved in Stx but not MPR transport [60], and knockdown of an oxysterol-binding protein (ORP5) reduces retrograde transport of the cation independent MPR but not of Stx [61], for review, see [53], [62]{Pfeffer, 2009 121/id}. As shown here, we also found no change in the distribution of MPRs after knockdown of annexin A1, indicating that the recycling route of those receptors does not involve annexin A1. These results, in addition to the lack of effect on ricin transport and on total sulfation, support the idea of a specific role of annexin A1 in early endosome to Golgi transport of Stx. Moreover, we ensured that the localization of both TPST1 and TPST2, responsible for sulfation in the TGN, were not affected by knockdown of annexin A1, also confirming the specific role of annexin A1 in Stx transport and more specifically that it is not involved in the general function of the Golgi apparatus in general (i.e. sulfotransferases localisation, MPR recycling, retrograde transport of ricin).

It has been shown that inhibition of PKCδ induces formation of an actin coat surrounding early endosomes, preventing sorting of cargo such as EGFR from this compartment [63], [64]. A similar endosomal accumulation has also been shown for Stx after knockdown or inhibition of PKCδ [15]. Interestingly, as we show here, annexin A1 knockdown resulted in an increased Stx transport to the Golgi which is blocked by PKCδ or cPLA_2_ inhibition. One might therefore speculate that annexin A1 acts in line with PKCδ and cPLA_2_. We also demonstrate the close proximity of annexin A1 with cPLA_2_α. Annexin A1 has been shown to inhibit cPLA_2_α [32], [43], therefore, even if the exact molecular mechanism remains to be further investigated, we propose that the negative regulatory role of annexin A1 on Stx transport is mediated by its inhibitory effect on cPLA_2_α. When annexin A1 is depleted, more cPLA_2_α might be free and active, inducing an increased transport of Stx to the Golgi apparatus. It is interesting to note that Stx appears to induce dissociation of the cPLA_2_α/annexin A1 complex, leading to activation of cPLA_2_α, thereby stimulating its own transport from endosomes to the Golgi apparatus.

Annexin A1 and A2 are structurally closely related, and could perhaps conduct compensatory actions within the cell. In cells derived from annexin A1 knockout mice, it was reported that the annexin A2 protein is upregulated but not taking over all annexin A1 functions [65], [66]. Our data are consistent with a non-overlapping role of annexin A1 and A2. Moreover, in the cells studied here, we did not observe any upregulation of annexin A1 or A2 when the other is knocked down, neither at the mRNA nor at the protein level. Annexin A2 has been found to be associated with clathrin on endosomes [26]. Earlier studies have also shown that depletion of annexin A2 by siRNA results in repositioning of transferrin-positive endosomes colocalizing with Rab11 and an inhibition of EGF degradation [27], [28]. Moreover, electron microscopy pictures showed that clathrin is accumulating at endosomal buds in annexin A2 depleted cells [27]. These data implicate annexin A2 in the clathrin-dependent transport from early endosomes, and are in agreement with an effect on both the endocytic recycling pathway and the degradative pathway. Interestingly our data indicating a positive effect of annexin A2 on Golgi transport of Stx are in line with the literature, since Stx transport to the Golgi apparatus has also been shown to be dependent on endosomal clathrin [4] and Rab11 [60].

The accepted role of the annexins is to be organizers of membrane domains, at the plasma membrane and at endosomal compartments. Therefore, drawing a clear picture of the highly dynamic events involving annexins is of crucial importance in the field of membrane trafficking. As shown here, toxins and specifically Stx, prove to be useful tools to shed light on cellular mechanisms involving annexin proteins. We show here that annexin A1 specifically inhibits retrograde transport of Stx from endosomes to the Golgi apparatus, and our data suggest that annexin A1/cPLA_2_α interactions are involved in this process. Very recently a new role for cPLA_2_α in vesiculation of cholesterol enriched endosomes containing GPI-anchored proteins was reported [51]. Those data together with the results reported here shed light on the complexity of the fine tuning needed for regulation of trafficking events. Interestingly, another member of the annexin family, annexin A6 has been shown to be involved in a cholesterol-dependent recruitment of cPLA_2_ to the Golgi apparatus and thereby inhibiting cPLA_2_ activity [67]. More recently it has been shown that this inhibitory effect of annexin A6 towards cPLA_2_ strongly affects function of t-SNARE proteins involved in the post-Golgi exocytic pathway [68]. Thus, there may be a more general role for different members of the annexin family in controlling the activity of cPLA_2_. However, further studies should be performed in order to determine the exact molecular details.

## Materials and Methods

### Antibodies and reagents

The mouse anti-annexin A2 (clone 5), anti-annexin A1 (clone 29) and anti-Hsp90 antibodies were from BD Biosciences (NJ, USA). The mouse monoclonal anti-annexin A2 antibody termed HH7 used in confocal microscopy has been described previously [69]. The mouse anti-Stx (clones 3C10 and 13C4) were from Toxin Technology (FL, USA) and the rabbit anti-ricin antibodies were from Sigma-Aldrich. The HRP-, Cy2-, Cy3- and Cy5-conjugated secondary antibodies were from Jackson Immunoresearch (PA, USA). The sheep anti-TGN46 and the rabbit anti-cPLA_2_ (clone N-216) were from Serotec (NC, USA), and Santa Cruz (CA, USA) respectively. Protein A sepharose was from Amersham Biosciences, (Buckinghamshire, UK). Na_2_^35^SO_4_ and [^3^H]leucine were from Hartmann Analytic (Braunschweig, Germany). Shiga holotoxin was provided by Dr. J. L. Kozlov (Academy of Sciences of Russia, Moscow, Russia) and Dr. J. E. Brown (USAMRIID, Fort Detrick, MD, USA). Ricin was from Sigma-Aldrich. Other reagents used were from Sigma-Aldrich if nothing else is stated.

### Plasmids

The plasmid expressing ShigaB-sulf2 was a gift from Dr. B. Goud (Institut Curie, Paris, France) and ShigaB-sulf2 was prepared as described elsewhere [70]. Ricin-sulf1 was produced from a plasmid expressing the ricin A-chain with one available sulfation site and reconstituted with the ricin B-chain after purification, as described earlier [39]. The annexin A2-CFP construct has been described previously [71] and was a kind gift from Dr. U. Rescher. The annexin A1-GFP and annexin A1-Y21F [22] were gifts from Dr. C. Futter (Institute of Ophthalmology, UK).

### Cell lines and Transfections

Human cervical carcinoma HeLa cells (obtained from ATCC/LGC) were maintained at 37°C in 5% CO_2_ in Dulbecco's Modified Eagle medium, DMEM (Invitrogen, Carlsbad, CA, USA), supplemented with 10% (w/v) fetal bovine serum (PAA Laboratories, Linz, Austria), 100 U/mL penicillin (Invitrogen) and 100 μg/mL streptomycin (Invitrogen). Cells were routinely seeded 24 h prior to transfection or other experimental treatments. To silence annexin A1 and A2 protein expression the following target siRNAs were purchased from Dharmacon (Lafayette, CO, USA); AnxA1-1: 5′-GAAGTGCGCCACAAGCAAA-3′, AnxA1-2: 5′-CAAAGGTGGTCCCGGATCA-3′, AnxA2-1: 5′-GGTCTGAATTCAAGAGAAA-3′, AnxA2-2: 5′-AAAACCAGCTTGCGAATAA-3′. Cells were transiently transfected for 5 h with 25 nM of annexin siRNAs or ON-TARGETplus control oligos using DharmaFECT^TM^ 1 (Dharmacon), according to the manufacturer's protocol. Cells were then grown in media containing serum and antibiotics for 48 or 72 h. Plasmid transfections in HeLa cells were performed using FuGENE-6 (Roche Diagnostics) according to the protocol of the manufacturer. Empty vectors were introduced to control cells. Cells were then grown in media containing serum and antibiotics for 24 h.

### Immunofluorescence

Cells used for immunofluorescence staining were routinely rinsed in PBS, fixed in 10% formalin in PBS (Sigma-Aldrich) for 15 min, permeabilized with 0.1% Triton X-100 in PBS for 5 min and blocked with 10% FBS in PBS for 30 min before incubation with primary and secondary antibodies. Coverslips were mounted in Mowiol or Prolongold with DAPI (Invitrogen) and images were acquired with the laser scanning microscope (LSM) 510 Meta from Carl Zeiss (Oberkochen, Germany).

### Sulfation of ShigaB-sulf2 and ricin-sulf1

In order to study endosome-to-Golgi transport of Stx and ricin, modified toxin molecules containing available sulfation sites were used [39], [70]. HeLa cells transfected with siRNA against annexin A1 or A2 for 72 h were starved in a sulfate-free medium containing 0.2 mCi/mL Na_2_^35^SO_4_ for 3 h at 37°C. When stated, cells were incubated during the last 30 min, or 60 min for MAFP, with 30 μM of SB203580 (MAPK p38 inhibitor), 2.5 μM of rottlerin (PKCδ specific inhibitor), 5 μM of ONO-RS-082 (cPLA_2_ inhibitor), 15–50 μM of MAFP (cPLA_2_ inhibitor) or 0.3% (v/v) of DMSO as control. Toxin constructs (ShigaB-sulf2 or ricin-sulf1) were then added in the range of 0.2–1 μg/mL and incubated at 37°C for the time indicated for each experiment. To measure the amount of radioactively labeled ShigaB-sulf2 or ricin-sulf1, cells were lysed in 0.1 M NaCl, 10 mM Na_2_HPO_4_, 1 mM EDTA, 1% Triton X-100 and 60 mM *n*-octyl-glucopyranoside, supplemented with a mixture of protease inhibitors (Roche Molecular Biochemicals, Mannheim, Germany), pH 7.4. Cell lysates were centrifuged for 10 min at 6000 rpm at 4°C to remove the nuclear fraction, and toxin contructs were immunoprecipitated with protein A sepharose beads coated with mouse anti-Stx (3C10) or ricin antibody overnight at 4°C. Beads were washed twice in PBS containing 0.35% Triton-X 100 and resuspended in sample buffer. Proteins were separated on a SDS-PAGE gel, blotted onto a PVDF membrane and analyzed by autoradiography using the Quantity One® 1-D Analysis Software 4.6.5 (Bio-Rad Laboratories Inc., CA, USA). Knockdown of annexin A1 or A2 were validated by immunoblotting within each experiment. For this, total cell lysates remaining after immunoprecipitation were subjected to SDS-PAGE and transferred onto a PVDF membrane. Before incubation with primary antibodies overnight at 4°C, blocking was performed with 3% dry milk in PBS for 30 min at RT. Following incubation with HRP-conjugated secondary antibodies, enhanced chemiluminescence was used for signal detection. Alternatively, the Odyssey Infrared Imaging System from LI-COR (NE, USA) was used after incubation with IRDye infrared linked secondary antibody (LI-COR) according to manufacturer instructions. As control of endogenous sulfotransferase activity, the radioactivity in trichloroacetic acid precipitated proteins from total cell lysates was measured using a Packard Tri-Carb Liquid Scintillation Analyzer 2100TR β-counter (PerkinElmer).

### Proximity ligation assay

HeLa cells were fixed directly as described in the immunofluorescence section or washed once in warm Hepes buffered medium and left 20 min at 37°C before addition or not of ShigaB-sulf2 construct at about 1 μg/mL for 10 min. The proximity between cytoplasmic PLA2 and annexin A1 or annexin A2 has been evaluated using the proximity ligation assay kit, Duolink^TM^ (Olink Bioscience, Uppsala, Sweden) [48], [72], according to the instructions from the manufacturer. Briefly, primary antibodies raised against annexin A1 from BD Biosciences and cPLA_2_ from Santa Cruz were used either alone as negative control or in combination. Cells were then incubated with PLUS and MINUS secondary PLA probes conjugated with oligonucleotides against both rabbit and mouse IgG. The two complementary oligonucleotides have then been hybridized, ligated and amplified by the provided polymerase. Detection was achieved with complementary, fluorescently labeled oligonucleotides using the 563 detection kit from Olink Bioscience, resulting in red fluorescence signals when the targeted proteins are closer than 40 nm. Fluorescence spots obtained were counted automatically for at least 40 cells per condition using ImageJ software and the average number of spots per cell was calculated.

### Statistical Analysis

Statistical comparisons were made by using the Student's t-test. A value of *p*<0.05 was regarded as a statistically significant change. The ImageJ software was used for the quantification analysis of confocal pictures and for the counting of spots in proximity ligation assay. Photoshop CS2 was used to prepare images.

See Text S1 for details of experiments shown in Supplementary figures.

## Supporting Information

Figure S1 **Quantification of annexin A1 and A2 mRNA levels after siRNA treatment.** mRNA from HEp-2 cells transfected with indicated siRNA was extracted and cDNA was synthesized by RT-PCR. The relative amounts of annexin A1 or A2 cDNA were determined by real-time PCR and normalized to the level of annexin A1 or A2 cDNA in control siRNA treated cells. The black and white bars represent annexin A1 and A2, respectively. Data presented are the result from one representative experiment, error bars indicating average deviation between duplicates.
(TIF)

Figure S2 **Annexin A1 and A2 knockdown alter endosome-to-Golgi transport of ShigaB in HEp-2 cells.** Quantative data from protein sulfation plotted as percentages of control values. HEp-2 cells transfected with indicated siRNA against annexin A1 or A2 were incubated with ShigaB. The white and black bars represent immunoprecipitated sulfated ShigaB detected by autoradiography, and total protein sulfation, respectively. Data presented are the average of 3–8 independent experiments, each performed in parallel, error bars indicating standard error of the mean; *p<0.05, **p<0.005 indicates statistically significant change.
(TIF)

Figure S3 **Colocalization of annexin A1 or A2 with Stx.** HeLa cells were fixed and stained for Annexin A1 or A2 (red) after 20 or 40 min incubation with Stx-K3 prelabeled with Alexa-488 (green). Due to major differences in staining intensities of annexin A1 in nuclei compared to other cellular areas, inserts were created with increased red color intensity to show partial colocalization between annexin A1 and Stx at the plasma membrane. Scale bar, 10 μm.
(TIF)

Figure S4 **Distribution of MPRs and TPST in annexin A1 depleted cells.** (A) To visualize the localization of MPRs, cells were fixed, permeabilized and immunostained with a sheep anti-TGN antibody in combination with a mouse monoclonal anti-CD or -CI MPR antibody. In (B), cells were transfected with an EGFP-TPST2 expression plasmid 48 h after transfection with siRNA. TGN was stained as in (A). Scale bars 10 μm. The relative colocalization of MPR46, MPR300 or EGFP-TPST2 with TGN46 positive structures was quantified by ImageJ software. Graphs represent the average from 25 cells plotted as percentage of total fluorescence for each marker, for one representative experiment (n = 3), where error bars indicate standard deviation.
(TIF)

Figure S5 **Binding and endocytosis of Stx in Annexin A1 depleted cells.** Stx binding and endocytosis following annexin A1 knockdown. HeLa cells transfected with siRNA as indicated for 72 h were incubated with ∼ 0.5 nM biotin-Stx for the indicated timepoints. Endocytosed and total cell-associated toxin were quantified by electrochemiluminescence and compared to control siRNA treated cells. Data shown are the results from one representative experiment (n = 3) where error bars indicate standard deviation between triplicates from one sample.
(TIF)

Figure S6 **Reduced ShigaB sulfation in response to cPLA2 inhibitor MAFP.** HeLa cells were starved in the presence of radioactive sulfate for 2 h before addition of MAFP at the indicated concentrations. After 1 hour, ShigaB-sulf2 was added, and the incubation continued for an additional 45 minutes. Cells were lysed, ShigaB immunoprecipitated, separated by electrophoresis and analyzed by autoradiography. The total amount of sulfated protein was analyzed by TCA precipitation. The autoradiography (upper panel) shows results from one sulfation experiment performed in triplicate (duplicate for 50 µM MAFP), and the bar graph shows the quantification plotted as percentages of control values with error bars indicating standard deviation. The experiment was repeated once with similar results.
(TIF)

Figure S7 **Absence of proximity for annexin A1 and GFP.** Close proximity of annexin A1 with expressed GFP was evaluated using the proximity ligation assay from Duolink as described in the materials and methods section. Fixed and permeabilized cells were incubated with the annexin A1 and GFP antibodies. Scale bar is 20 μm. The panel shows a representative GFP expressing cell with very few dots comparable to the surrounding untransfected cells.
(TIF)

Text S1 **Methods.**
(DOCX)
