# Supplementary figures and images for: Annexin A1 and A2: Roles in Retrograde Trafficking of Shiga Toxin
Source: PLoS One. 2012 Jul 6;7(7):e40429. doi: 10.1371/journal.pone.0040429 (PMC3391278; doi:10.1371/journal.pone.0040429)

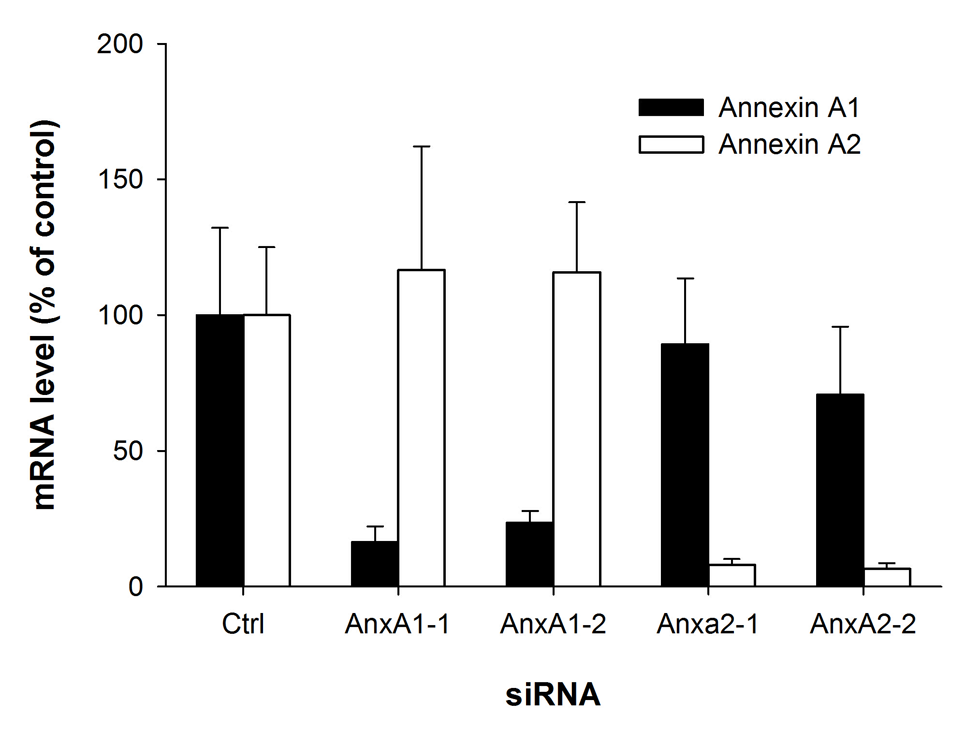

Supplement: Figure S1 — Quantification of annexin A1 and A2 mRNA levels after siRNA treatment. mRNA from HEp-2 cells transfected with indicated siRNA was extracted and cDNA was synthesized by RT-PCR. The relative amounts of annexin A1 or A2 cDNA were determined by real-time PCR and normalized to the level of annexin A1 or A2 cDNA in control siRNA treated cells. The black and white bars represent annexin A1 and A2, respectively. Data presented are the result from one representative experiment, error bars indicating average deviation between duplicates. (TIF) [file pone.0040429.s001.tif]

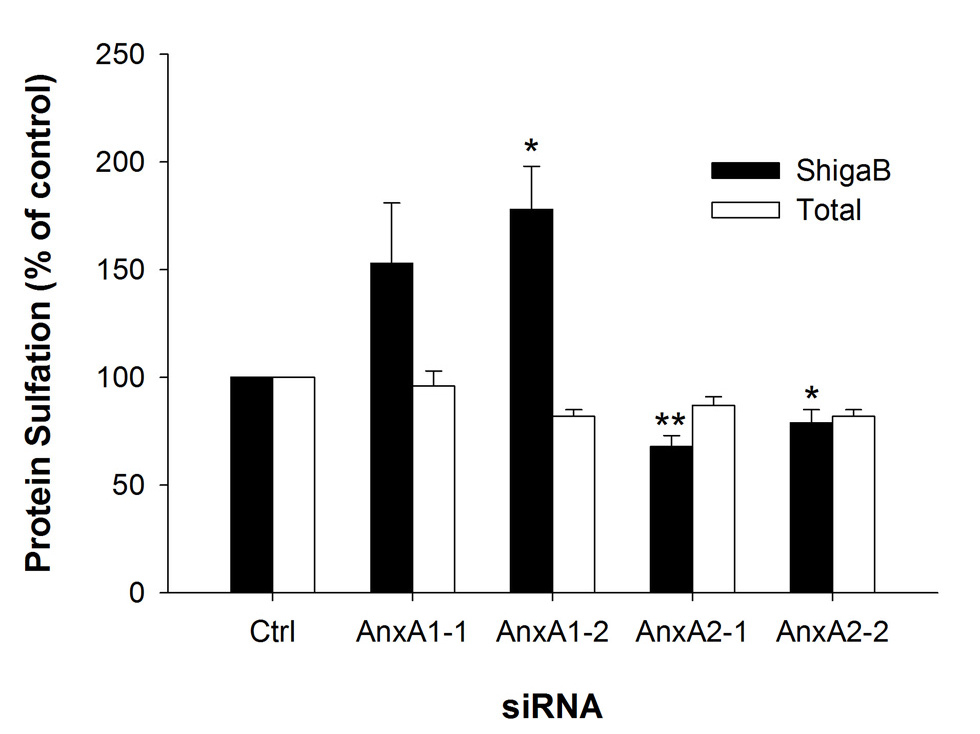

Supplement: Figure S2 — Annexin A1 and A2 knockdown alter endosome-to-Golgi transport of ShigaB in HEp-2 cells. Quantative data from protein sulfation plotted as percentages of control values. HEp-2 cells transfected with indicated siRNA against annexin A1 or A2 were incubated with ShigaB. The white and black bars represent immunoprecipitated sulfated ShigaB detected by autoradiography, and total protein sulfation, respectively. Data presented are the average of 3–8 independent experiments, each performed in parallel, error bars indicating standard error of the mean; *p<0.05, **p<0.005 indicates statistically significant change. (TIF) [file pone.0040429.s002.tif]

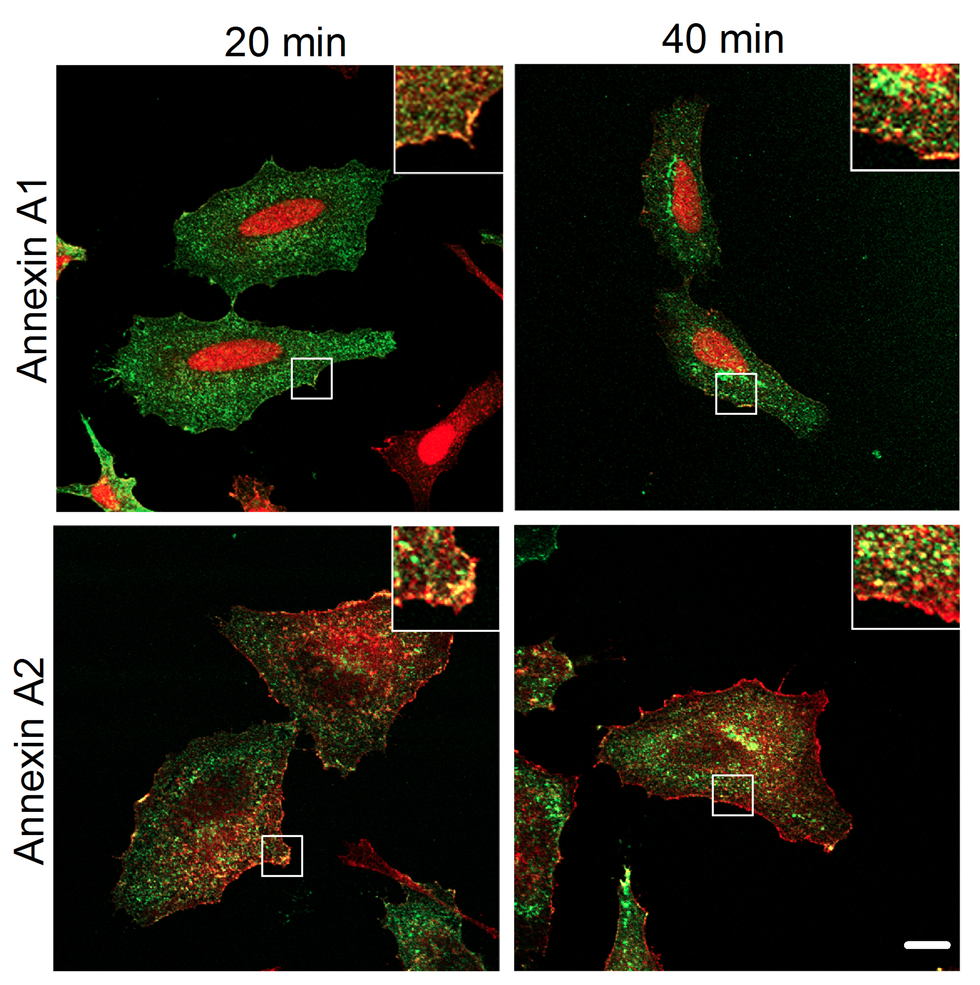

Supplement: Figure S3 — Colocalization of annexin A1 or A2 with Stx. HeLa cells were fixed and stained for Annexin A1 or A2 (red) after 20 or 40 min incubation with Stx-K3 prelabeled with Alexa-488 (green). Due to major differences in staining intensities of annexin A1 in nuclei compared to other cellular areas, inserts were created with increased red color intensity to show partial colocalization between annexin A1 and Stx at the plasma membrane. Scale bar, 10 μm. (TIF) [file pone.0040429.s003.tif]

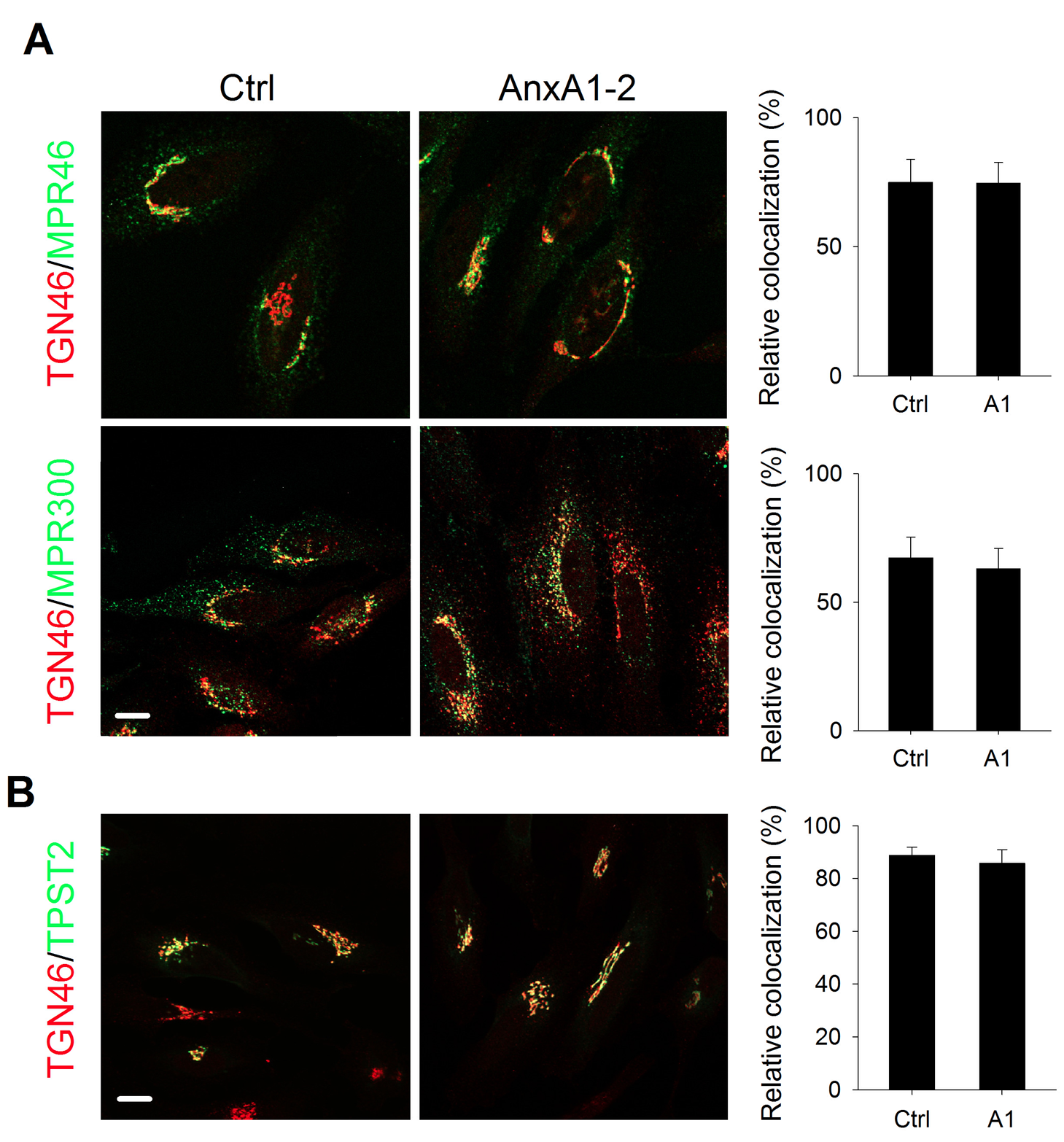

Supplement: Figure S4 — Distribution of MPRs and TPST in annexin A1 depleted cells. (A) To visualize the localization of MPRs, cells were fixed, permeabilized and immunostained with a sheep anti-TGN antibody in combination with a mouse monoclonal anti-CD or -CI MPR antibody. In (B), cells were transfected with an EGFP-TPST2 expression plasmid 48 h after transfection with siRNA. TGN was stained as in (A). Scale bars 10 μm. The relative colocalization of MPR46, MPR300 or EGFP-TPST2 with TGN46 positive structures was quantified by ImageJ software. Graphs represent the average from 25 cells plotted as percentage of total fluorescence for each marker, for one representative experiment (n = 3), where error bars indicate standard deviation. (TIF) [file pone.0040429.s004.tif]

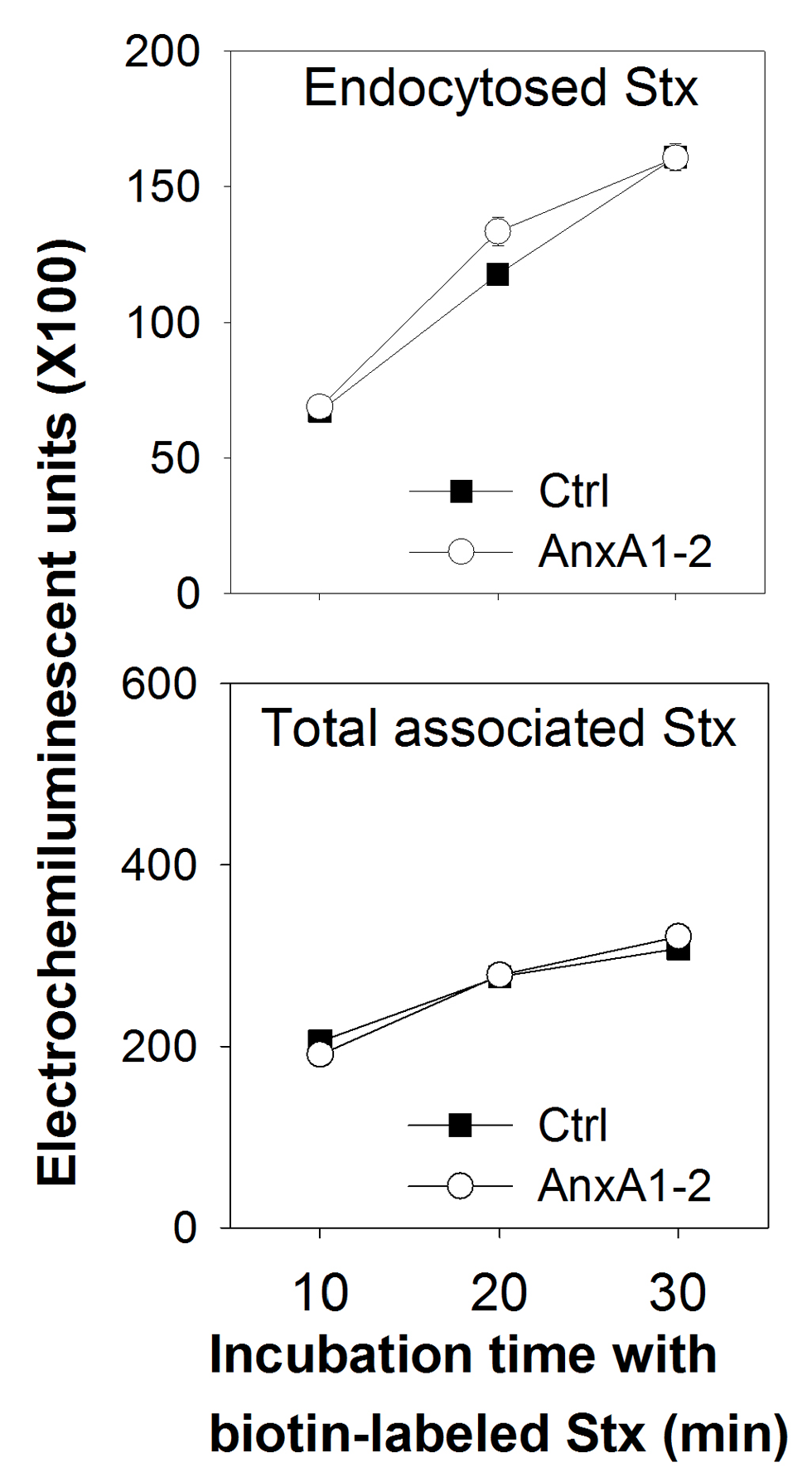

Supplement: Figure S5 — Binding and endocytosis of Stx in Annexin A1 depleted cells. Stx binding and endocytosis following annexin A1 knockdown. HeLa cells transfected with siRNA as indicated for 72 h were incubated with ∼ 0.5 nM biotin-Stx for the indicated timepoints. Endocytosed and total cell-associated toxin were quantified by electrochemiluminescence and compared to control siRNA treated cells. Data shown are the results from one representative experiment (n = 3) where error bars indicate standard deviation between triplicates from one sample. (TIF) [file pone.0040429.s005.tif]

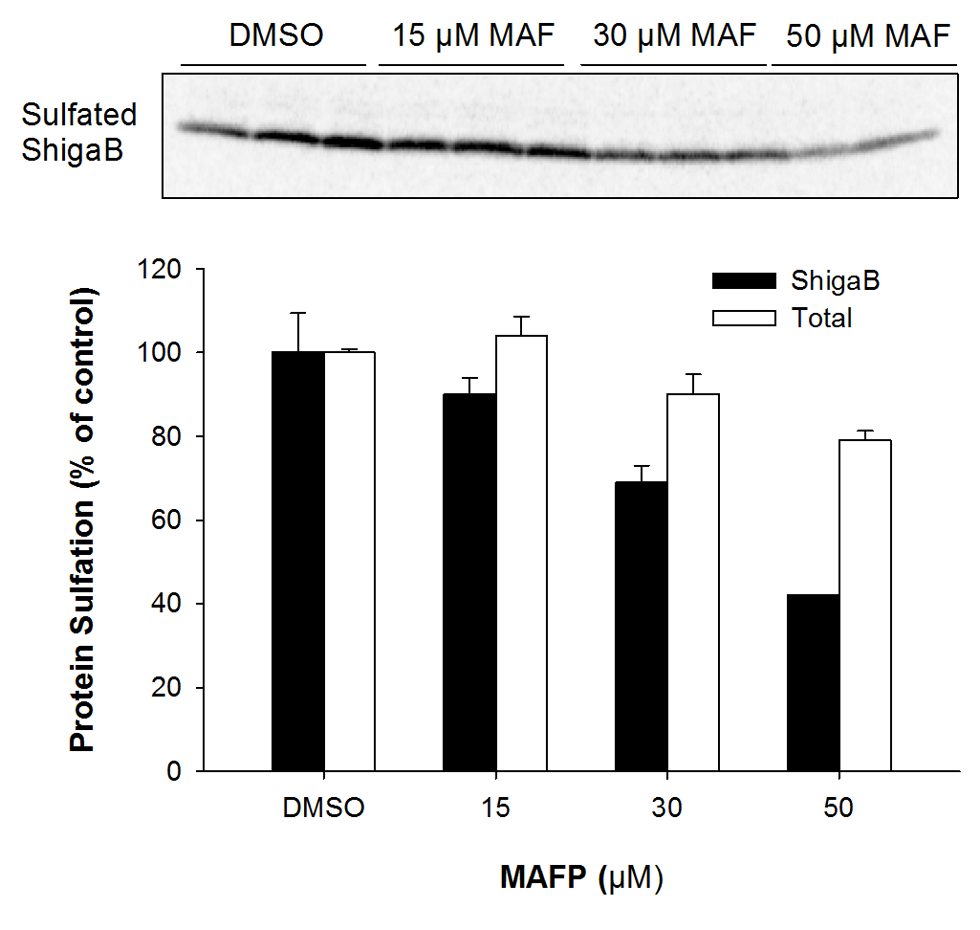

Supplement: Figure S6 — Reduced ShigaB sulfation in response to cPLA2 inhibitor MAFP. HeLa cells were starved in the presence of radioactive sulfate for 2 h before addition of MAFP at the indicated concentrations. After 1 hour, ShigaB-sulf2 was added, and the incubation continued for an additional 45 minutes. Cells were lysed, ShigaB immunoprecipitated, separated by electrophoresis and analyzed by autoradiography. The total amount of sulfated protein was analyzed by TCA precipitation. The autoradiography (upper panel) shows results from one sulfation experiment performed in triplicate (duplicate for 50 µM MAFP), and the bar graph shows the quantification plotted as percentages of control values with error bars indicating standard deviation. The experiment was repeated once with similar results. (TIF) [file pone.0040429.s006.tif]

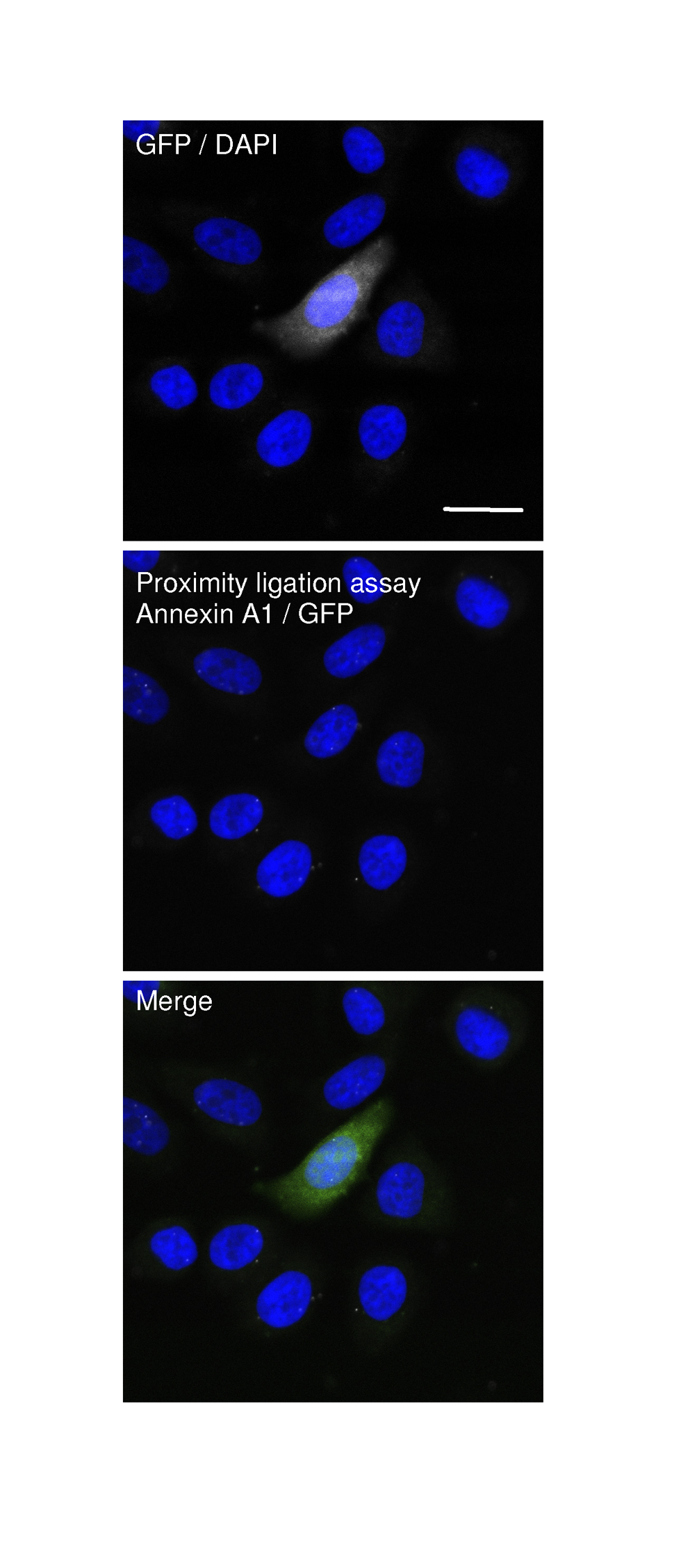

Supplement: Figure S7 — Absence of proximity for annexin A1 and GFP. Close proximity of annexin A1 with expressed GFP was evaluated using the proximity ligation assay from Duolink as described in the materials and methods section. Fixed and permeabilized cells were incubated with the annexin A1 and GFP antibodies. Scale bar is 20 μm. The panel shows a representative GFP expressing cell with very few dots comparable to the surrounding untransfected cells. (TIF) [file pone.0040429.s007.tif]
