# Supplementary material for: Annexin A1 and A2: Roles in Retrograde Trafficking of Shiga Toxin
Source: PLoS One. 2012 Jul 6;7(7):e40429. doi: 10.1371/journal.pone.0040429 (PMC3391278; doi:10.1371/journal.pone.0040429)
Supplement: Text S1 — Methods. (DOCX) [file pone.0040429.s008.docx]

**Supplementary text S1**

**Methods**

**Real time RT-PCR**

The efficiency of the siRNA oligos were initially tested by Real-time RT PCR using the following sets of primers; AnxA1 forward 5´-AACTTCGCAGAGTGTTTCAGAAA-3´ and reverse 5´- CGATAGCTGTGAGGCATTTC-3´; AnxA2 forward 5´-TGACTCCATGAAGGGCAAG-3´ and reverse 5´-TAGTGTCTTGCTGGATATAATAG-3´, by the procedures described previously (Utskarpen *et al.*, 2007). Briefly, Aurum Total RNA Mini Kit and iScript cDNA Synthesis Kit (Bio-Rad Laboratories) were used for RNA isolation and cDNA synthesis on HEp-2 cells treated with indicated siRNA. The following program was used on a LightCycler 480 Real-Time PCR System using LightCycler 480 SYBR green 1 Master mix (Roche): Preincubation 5 min 95 °C, amplification 45 cycles (10 s denaturation 95 °C, 20 s annealing 60 °C and 10 s extension 72 °C). TATA box binding protein was used as an internal control and the software LightCycler 480 Relative Quantification Software (Roche) was used for quantification.

**Sulfation of ShigaB-sulf2 in HEp-2 cells**

HEp2 cells were used in the exact same procedure as HeLa cells to determine Stx transport to the Golgi apparatus (see the Sulfation procedure in materials and methods section).

**Colocalization of annexin A1 or A2 with Stx**

HeLa cells were rinsed in warm Hepes buffered medium and incubated with StxB-K3-488 at about 1 μg/mL at 37°C for 20 or 40 min. StxB-K3 produced as earlier described (Raa et al., 2009) was prelabeled with the Alexa-488 microscale protein labeling kit (Molecular Probes). To visualize the localization of annexin A1 or A2, cells were fixed, permeabilized and immunostained with a mouse monoclonal antibody against annexin A1 or A2 (HH7). Confocal images were acquired as described in the material and methods section.

**Colocalization of MPRs and TPST in annexin A1 depleted cells**

HeLa cells were transfected for 72 h with control, or either annexin A1 siRNA. Mouse monoclonal anti-CD MPR antibody, given by Dr. R. Pohlmann (Münster, Germany) and anti-CI MPR from Abcam (Cambridge, UK), have been used in combination with sheep anti-TGN. Localisation of TPST-1 and TPST-2 were assessed by overexpressing the plasmids TPST1-EGFP and TPST2-EGFP kindly provided by Dr. L. Johannes (Institut Curie, France) (Spooner *et al.*, 2008). The relative colocalization of MPR46, MPR300 or EGFP-TPST2 with TGN46 positive structures was evaluated as described in the materials and methods section.

**Endocytosis of Stx**

The procedure for assaying cellular binding and uptake of Stx followed the protocol already described (Lauvrak *et al.*, 2006; Torgersen *et al.*, 2007). Briefly, HeLa cells transfected with siRNA against annexin A1 (72 h) or non targeting siRNA were incubated with biotin-labeled Stx (biotin-Stx) for 10, 20 or 30 min at 37°C in Hepes buffered media. Cells were then washed in a cold Hepes buffer (0.14 M NaCl, 2 mM CaCl_2_, 20 mM Hepes; pH 8.6). To distinguish between endocytosed and total cell associated biotin-Stx, half of the cells were treated for 30 min on ice with 0.1 M MESNa, which reduces the biotin disulfide bond of surface bound toxin. This allows us to measure internalized toxin. The other half was used to determine total cell-associated biotin-Stx. Cells were lysed (100 mM NaCl, 5 mM MgCl_2_, 1% (v/v) Triton X-100, 50 mM Hepes, 60 mM *n*-octylglucopyranoside) for 10 min and incubated with TAG^®^-labeled anti-Stx antibody (0.5 μg.mL^-1^) and streptavidin-coated magnetic beads (0.1 mg.mL^-1^, Invitrogen) with gentle shaking for 1.5 h. The amount of streptavidin-captured Stx complexed to the TAG^®^-labeled antibody was quantified by an M1R Analyzer (BioVeris Corporation).

**Proximity ligation assay**

HeLa cells were treated in the same way as described in the materiel and method section except that the cells were transfected with the GFP expressing plasmid (pEGFP-C2 from Clontech) 24h prior the assay using FuGENE-6 (Roche Diagnostics) according to the protocol of the manufacturer. The annexin A1 and GFP antibodies (BD Bioscience – clone 29 and Santa Cruz – sc-8334 repectively) were used to detect interaction events.
